# From illness to inactivity: Exploring the influence of physical diseases on youth Not in Education, Employment, or Training status in Europe: A systematic literature review

**DOI:** 10.1002/jad.12386

**Published:** 2024-08-08

**Authors:** Victoria Lindblad, Kristian Hay Kragholm, Pernille Skou Gaardsted, Line Elise Møller Hansen, Fie Falk Lauritzen, Dorte Melgaard

**Affiliations:** ^1^ Department of Gynecology, Pregnancy and Childbirth North Denmark Regional Hospital Hjoerring Denmark; ^2^ Department of Cardiology Aalborg University Hospital Aalborg Denmark; ^3^ Faculty of Clinical Medicine Aalborg University Aalborg Denmark; ^4^ Medical Library, Aalborg University Hospital Aalborg Denmark; ^5^ Department of Acute Medicine and Trauma Care Aalborg University Hospital Aalborg Denmark

**Keywords:** education, health, NEET, physical risk factors, systematic review, unemployment

## Abstract

**Introduction:**

In 2010, 33% of young Europeans (ages 15–29) were Not in Education, Employment, or Training (NEET), rising to 40 million by 2015. Those with disabilities or health challenges are 40% more likely to be NEET. Hence, we conducted a systematic search to identify health challenges as NEET risk factors.

**Methods:**

A systematic search was conducted across four databases on February 21, 2023, with an update on January 15, 2024. Data collected after 1980 were included. The main findings from this search concerning risk factors are summarized in a chart.

**Results:**

A total of 33,314 articles were screened, resulting in the inclusion of 32 articles in this review. The review identified multiple physical risk factors associated with NEET status, which were categorized into two primary domains: congenital conditions and birth‐related factors, for example, factors encompassed neonatal life in utero and experiences related to birth, and health conditions during childhood and adolescence, for example, survivors of childhood cancer and other severe health conditions during childhood and adolescents.

**Conclusions:**

Our findings highlight that varying congenital conditions and birth‐related factors as well as diseases from childhood to adulthood challenges or even hinder educational and job market participation, this emphasizing the importance of targeted support for children facing health challenges. These findings highlight the immediate requirement for comprehensive interventions specifically designed for children and adolescents who are for example, preterm, have experienced severe illness, or are coping with chronic diseases. These interventions should address the challenges encountered by youth in NEET. However, limited evidence on the impact of health conditions on NEET status underscores the necessity for further research into both short‐ and long‐term effects.

## 1 INTRODUCTION

In recent decades, policymakers globally have increasingly focused on the issue of young individuals disengaged from education or employment. This has led to various intervention initiatives amidst high political expectations. The term “NEETs” refers to young people aged 15–29 years who are Not Engaged in Education, Employment, or Training (OECD, 2024). In 2015, the number of NEETs in The Organization for Economic Cooperation and Development (OECD) countries was approximately 40 million (OECD, 2016). Of these, over two‐thirds (28 million young people) are inactive, meaning they are not even seeking work (OECD, 2016). Research revealed that in 2010, there were about 14 million NEETs in Europe, representing a significant 33% of the total young European population (Eurofound et al., 2012). At the EU level, NEETs are considered one of the most problematic groups in the context of youth unemployment (Eurofound et al., 2012). Young people with NEET status are more likely to encountering somatic or mental health challenges later in life compared to their peers. When young individuals are not actively engaged in education or employment, the repercussions for their life trajectory are significant. These encompass deteriorating health, heightened mental health issues, diminished quality of life, and an increased susceptibility to social isolation, poverty, and involvement in criminal activities due to their disengagement from education or employment pathways (Raghupathi & Raghupathi, 2020; Zajacova & Lawrence, 2018; Zimmerman & Woolf, 2014).

Understanding the specific challenges faced by young people in the school system and during the transition to the labor market is crucial for initiating early support efforts. Research consistently shows that certain demographic groups are at a higher risk of becoming NEETs compared to others (Gariépy et al., 2022; Rahmani et al., 2024). Individuals with lower levels of education are three times more likely to be NEET than their counterparts with tertiary education (Eurofound et al., 2012). Geographical disparities, characterized by the rural‐urban divide, neighborhood effects, and unequal access to resources, underscore the complex and systemic nature of youth unemployment and education disengagement (Brattbakk & Wessel, 2013; Karyda & Jenkins, 2018). More recent studies shows that socioeconomic factors like parental separation, parents' low level of education or unemployment, poverty have an significantly impact on the risk for the young people to become NEET (Karhina et al., 2023; Yi̇ği̇t et al., 2023). Similarly, young people with an immigrant background have a 70% higher likelihood of NEET status compared to nationals (Eurofound et al., 2012). Family background also significantly shapes this trend (Eurofound et al., 2012). Mentally challenged young people also struggle to cope with school, which impacts their trajectory into the workforce (Tong et al., 2023) Moreover, those dealing with disabilities or health challenges are 40% more likely to be NEET than their peers in good health (Eurofound et al., 2012). The most recent scoping review found that poor health and disability increase the risk of becoming NEET, including back discomfort, fibromyalgia, and other musculoskeletal issues (Rahmani et al., 2024). However, there is limited focus on how physical factors, such as preterm birth, early‐onset chronic diseases, common childhood diseases, and impairment, influence the risk of becoming NEET. We chose to compile the literature through a systematic search process to ensure a comprehensive synthesis of existing research. Therefore, the purpose of this review is to investigate existing evidence of physical risk factors associated with NEET among young Europeans aged 15–29 years.

## 2 METHODS

The PRISMA‐ScR Checklist, as outlined by Tricco et al. (2018), guided the structuring of this article (2018). This systematic literature review is part of a comprehensive report comprising four reviews examining physical, geographical, psychological, and social factors influencing young individuals' completion of secondary education or entry into the labor market, leading to inclusion in the NEET group. The foundation for these four systematic literature reviews lies in a thorough literature search, detailed below.

### 2.1 Eligibility criteria

The search framework PCC (participants, concept, and context) approach was applied to establish clear inclusion criteria for this systematic literature review (Pollock et al., 2023). The inclusion criteria were studies including young individuals aged 15–29 years classified as NEETs according to Eurostat's definition (OECD, 2024). The term NEET referred to young individuals who were neither currently employed nor had entered the workforce after finishing compulsory education, encompassing those who had not commenced secondary school within the last 4 weeks or had not completed secondary education while not engaged in work or training activities. We included all types of physical health conditions or disabilities, starting from conditions conceived in utero, congenital conditions, birth‐related conditions, and those occurring during childhood and youth up to the age of 29, which may pose a risk for NEET status. The study utilized data collected in a European country after 1980, encompassing 43 countries on the European continent (OECD, 2024). It exclusively considered primary research featuring quantitative data, while mixed methods studies required discernible quantitative data. Articles written in English, German, Danish, Norwegian, and Swedish were incorporated. The decision to exclude certain languages was primarily driven on resource constraints. Limited translation resources and time necessitated a focus on the included languages, enabling a more efficient screening process. Experimental studies were excluded due to the focus on naturally occurring risk factors.

### 2.2 Information sources and search strategy

Medline, Embase, PsycINFO, and Scopus were systematically searched on February 21, 2023, and updated on January 15, 2024. Keywords in the search included young people or synonyms such as the acronym NEET Not in Employment, Education, or Training (NEET), opportunity youth, or disconnected youth, and terms related to work, employment, unemployment, education, and training. The search terms were combined with Boolean operators AND or OR (Grewal et al., 2016). Various search techniques were utilized, incorporating proximity operators, controlled vocabulary thesaurus terms such as medical subject headings (MeSH), and a free‐text exploration encompassing all synonyms and variations of the keyword. Furthermore, the searches were enhanced through forward citation searching in Citationchaser (Haddaway et al., 2021). The search strategies employed across all databases were peer reviewed by a specialist medical librarian outside the author group and are detailed in Appendix 1.

### 2.3 Selection process and data extraction

The selection process was divided into two rounds. Initially, all authors independently screened the studies based on title and abstract in Covidence®, excluding studies not conducted in a European country. The first round required only one author to make an exclusion decision. In the second round, the authors independently screened the records in Covidence® according to the inclusion criteria. This round required two authors to decide whether the studies should be included or excluded based on the title and abstract. Disagreements in the screening process were discussed and resolved through consensus among all authors. Authors V. L. and D. M. collaborated on the full‐text screening, with disagreements resolved by consensus within the author group. Information about author(s), year of publication, title, aim(s) of the study, participants (number, age, and nationality), study design, and main findings was extracted from the studies and charted. We used an inductive methodology to categorize the findings based on patterns and themes identified in the included studies. This approach allowed the data to guide the development of our categorization. In cases where multiple studies addressed the same physical health concern, we condensed and presented the results collectively in the results section (Pollock et al., 2023).

## 3 RESULTS

**Figure 1:**
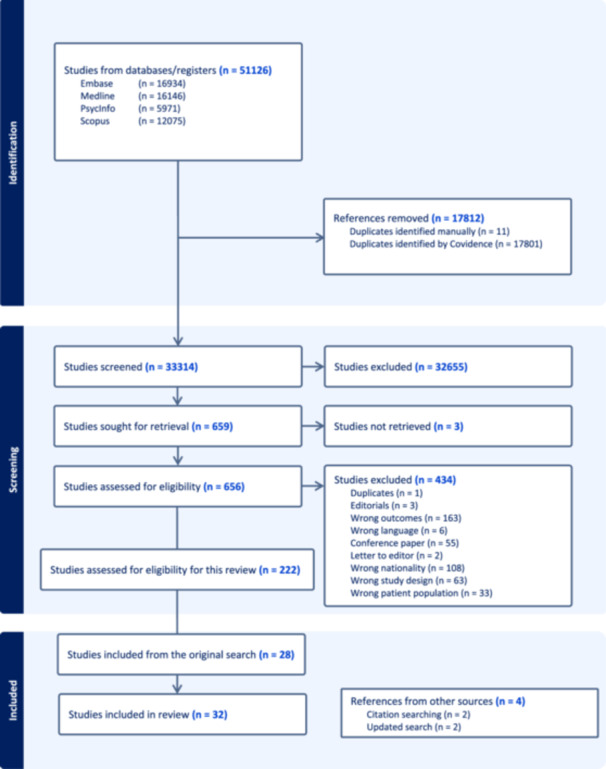
Flow diagram of screening process.

The authors diligently conducted a thorough literature search using the prescribed methods to identify relevant articles. Initially, 51,126 studies were identified through the search strategy. After removing duplicates, 33,314 studies underwent individual screening based on title and abstract, resulting in 32,655 being deemed irrelevant. Subsequently, 659 studies underwent full‐text screening, and 434 were excluded. Three studies could not be retrieved. Consequently, 222 studies met the criteria for consideration, of which 28 articles were deemed relevant to this study and yielded extractable data consistent with the inclusion criteria. An additional two studies were uncovered through citation searches, with an additional two included from the updated search Figure 1.

This literature review encompasses a total of 32 studies, including a substantial cohort of 7,067,507 participants. The population sizes in the studies varied from a minimum of 56 to a maximum of 2,454,862. Among these, 12 studies were conducted within the past 4 years (Baughan et al., 2023; Boros et al., 2022; Chen et al., 2021; Dionisi‐Vici et al., 2022; Fleming, McLay, et al., 2020; Hövels‐Gürich et al., 2023; Johansson et al., 2022; Kessel et al., 2022; Liu et al., 2023; Murray et al., 2020; Pedersen et al., 2023; Visnick et al., 2023) while another 14 were conducted between 2010 and 2020 (Abebe et al., 2019; Ayorech et al., 2019; Bania et al., 2019; Díaz‐mendoza et al., 2015; Dommergues et al., 2010; Fararouei et al., 2010; Fleming, et al., 2019a, 2019b; Frobisher et al., 2017; Glatz et al., 2017; Homlong et al., 2013; Lallukka et al., 2019; Mikkonen et al., 2018; Ridder et al., 2013). Notably, there were only six studies published between 2000 and 2010 that investigated the physical impact on the risk of young individuals becoming NEET (Bjerkedal et al., 2006; Calsbeek et al., 2006; Cooke, 2004; Haberal et al., 2000; Kristensen & Bjerkedal, 2004; Kristensen et al., 2005). Remarkably, no studies were identified before the year 2000.

As documented in Table 1 half of the studies originate from Scandinavian countries with seven conducted in Norway (Abebe et al., 2019; Bania et al., 2019; Bjerkedal et al., 2006; De Ridder et al., 2013; Homlong et al., 2013; Kristensen, 2004, 2009), four in Sweden (Chen et al., 2021; Glatz et al., 2017; Johansson et al., 2022; Liu et al., 2023), three in Finland (Fararouei et al., 2010; Lallukka et al., 2019; Mikkonen et al., 2018), and two in Denmark (Kessel et al., 2022; Pedersen et al., 2023). The remaining studies represent a diverse range of locations, including six from Scotland (Baughan et al., 2023; Fleming et al., 2019a, 2019b; Fleming, McLay, et al., 2020; Murray et al., 2020; Visnick et al., 2023), four from the United Kingdom (Ayorech et al., 2019; Boros et al., 2022; Cooke, 2004; Frobisher et al., 2017), and one each from Germany (Hövels‐Gürich et al., 2023), Italy (Dionisi‐Vici et al., 2022), Spain (Díaz‐mendoza et al., 2015), France (Dommergues et al., 2010), the Netherland (Calsbeek et al., 2006), and Turkey (Haberal et al., 2000).

**Table 1:** Summary of study details and population (sorted after publication year).

References	Title	Aim(s) of the study	Participants; number, age, and nationality	Study design	Main findings
Baughan et al. (2023)	Educational outcomes in childhood cancer survivors: A Scotland‐wide record‐linkage study of 766,217 schoolchildren	Investigate educational outcomes among schoolchildren with a previous cancer diagnosis compared to their peers	766,217 pupils versus 1313 with a previous cancer diagnosis – Scotland	Cohort	No significant associations with unemployment 6 months after leaving school for cancer overall
Hövels‐Gürich et al. (2023)	Sociodemographic parameters and noncardiac comorbidity related to self‐perceived quality of life in young adults after neonatal arterial switch operation for transposition of the great arteries	Evaluating the relation of noncardiac comorbidity and sociodemographic factors to physical and mental health‐related quality of life	92 young adults mean age 22.8 years – Germany	Cross‐sectional	The educational level was higher, and the rate of unemployment was double as high compared to the average population
Liu et al. (2023)	Educational outcomes in children and adolescents with type 1 diabetes and psychiatric disorders	Examine educational outcomes in adolescents with type 1 diabetes	2,454,862 born between 1973 and 1997 – Sweden	Cohort	Individuals with both type 1 diabetes and psychiatric disorders had lower odds of completing upper secondary high school compared with healthy individuals
Pedersen et al. (2023)	Children in Denmark with cerebral palsy rarely complete elementary school	How children with cerebral palsy (CP) perform in the school system and which factors are associated with school performance	463,126 children born from 1997 to 2003. Eight hundred and eighteen children with cerebral palsy (CP) (41% females) and 417,731 (49% females) without CP – Denmark	Cohort	Children with CP have a high risk of not completing elementary school. Children with CP achieve lower overall grades than children without CP
Visnick et al. (2023)	Educational and employment outcomes associated with childhood traumatic brain injury in Scotland: A population‐based record‐linkage cohort study	Compare the educational and employment outcomes of schoolchildren previously hospitalized for traumatic brain injury (TBI) with their peers	766,244 children. Four thousand seven hundred and eighty‐eight (0.6%) had a history of hospitalization for TBI – Scotland	Cohort	Compared to their peers, children previously hospitalized for TBI had and lower academic attainment post injury. However, no significant differences were observed in post school unemployment
Boros et al. (2022)	Juvenile Dermatomyositis: What comes next? Long‐term outcomes in childhood myositis from a patient perspective	Describe long‐term outcomes in juvenile dermatomyositis	84 adolescents, average age of respondents was 20.6 years. Time since diagnosis was 12.4 years. – United Kingdom and Ireland	Cohort combined with survey	Participants reported myositis adversely affecting academic results. They were twice as likely to be unemployed compared the general population
Dionisi‐Vici et al. (2022)	Work placement and job satisfaction in long‐term childhood cancer survivors: The impact of late effects	Describe the work placement and the perceived job and economic satisfaction of long‐term childhood cancer survivors	171 adolescents, age 18–29 years – Italy	Cross‐sectional	Being a survivor with severe comorbidities has a significantly negative impact on occupation and worsens the perception of satisfaction of economic situations
Johansson et al. (2022)	Celiac disease and upper secondary school achievement in Sweden A retrospective cohort study	Investigate school achievements in upper secondary school among adolescents with celiac disease	734,074 individuals, whereof 3257 (62% females) with celiac disease – Sweden	Cohort	Diagnosed celiac disease does not negatively affect school achievements in upper secondary school
Kessel et al. (2022)	Childhood‐onset retinal dystrophies reduces life‐time income by one‐third—An individual based socioeconomic analysis	Evaluate lifetime income, educational level and workforce participation in patients with childhood‐onset inherited retinal dystrophies (IRD)	515 patients with childhood‐onset IRD and without severe systemic comorbidities matched 1:4 to an age‐ and sex control sample of the background population – Denmark	Cohort	Few among those with childhood‐onset IRD were able to obtain high educational levels. Many were assigned a disability pension from early adulthood or were unemployed. Grade mark points from primary education were comparable, suggesting that the difference was not explained by intellectual differences between the groups
Chen et al. (2021)	Associations between multimorbidity patterns and subsequent labor market marginalization among refugees and Swedish‐born young adults—A nationwide registered‐based cohort study	Whether the association of multimorbidity patterns and Labor Market Marginalization (LMM) differs in refugee youth compared to Swedish‐born youth and identify the diagnostic groups driving this association	249,245 individuals between 20–25 years – Sweden	Cohort	Multimorbidity related similarly to LMM in refugees and Swedish‐born youth, but different diagnoses drove these associations
Fleming, McLay, et al. (2020)	Health, educational, and employment outcomes among children treated for a skin disorder: Scotland‐wide retrospective record linkage cohort study of 766,244 children	Compare health, educational, and employment outcomes of schoolchildren receiving medication for a skin disorder with peers	766,244 singleton children. Overall, 130,087 (17.0%) children were treated for a skin disorder – Scotland	Cohort	Despite increased hospitalization, school absenteeism, and special educational need, children treated for a skin disorder did not have poorer exam attainment or employment outcomes
Murray et al. (2020)	Association of gestational age at birth with risk of perinatal mortality and special educational need among twins	Identify the optimal gestation week for birth of twin infants by calculating the week of birth associated with the lowest risk of short‐term and long‐term adverse outcomes	43,133 twin infants (50.3% female) born at a gestational age of 34 weeks onward between January 1, 1980 and December 31, 2015 – Scotland	Cohort	Birth at any gestational age from 34 to 37 weeks was associated with increased risk of special educational need at school. Gestational age at birth was not associated with low attainment at school and birth before 37 weeks of gestation was not associated with unemployment. Birth at 39 weeks was associated with an increased risk of unemployment compared with that for birth at 37 weeks
Abebe et al. (2019)	Socioeconomic gradients and disability during the transition to young adulthood: A longitudinal survey and register study in Norway	Investigate trends and explanatory factors for socioeconomic inequalities associated with disability during the transition to young adulthood	2606 participants (56% females) – Norway	Cohort	Disabled adolescents have a significantly greater risk of achieving lower levels of education and are unemployed and over‐represented in welfare benefits during the transition to young adulthood
Ayorech et al. (2019)	Using DNA to predict educational trajectories in early adulthood	Tested for genetic influence on early adult decisions	5839 adolescents at 18 years of age – United Kingdom	Cohort	The gene EA3 was associated on average with a 51% reduction in the odds of pursuing full‐time employment. EA3 associations were attenuated when controlling for previous academic achievement and family socioeconomic status
Bania et al. (2019)	Not engaged in education, employment, or training (NEET) in an Arctic sociocultural context: The NAAHS cohort study	Prevalence and predictors of not engaged in education, employment, or training (NEET) status in a multicultural young adult population	5877 youth aged 15–16 years (83% of the total age cohort from all 87 municipalities participated). The follow‐up studies consisted of 3987 consent giving adolescents (68%), were 365 (9.2%) reported indigenous Sami ethnicity – Norway	Cohort	Musculoskeletal problems in male adolescents were associated with later NEET‐status
Fleming et al. (2019a)	Educational and health outcomes of children and adolescents receiving antiepileptic medication: Scotland‐wide record linkage study of 766 244 schoolchildren	Compare educational and health outcomes of children receiving antiepileptic medication versus peers	5314 children diagnosed with epilepsy and attending schools between 2009 and 2013 compared to 760,930 children not diagnosed with epilepsy – Scotland	Cohort	Children on antiepileptic medication were less likely, than their peers, to quit school before 16 years of age. Children on antiepileptic medication were more likely to be unemployed compared with peers
Fleming et al. (2019b)	Educational and health outcomes of children treated for asthma: Scotland‐wide record linkage study of 683 716 children	Questioned whether children treated for asthma suffer increased absenteeism, exclusion, special educational need, unemployment, hospital admission, mortality, and poorer attainment compared with peers	45,900 children diagnosed with asthma and attending schools between 2009 and 2013 compared to 6,327,816 children not diagnosed with asthma – Scotland	Cohort	A formal mediation analysis among girls with no recorded special educational need suggested that the effect of asthma on unemployment was likely to be an indirect effect mediated by absenteeism
Lallukka et al. (2019)	Determinants of long‐term unemployment in early adulthood: A Finnish birth cohort study	Social and health‐related determinants to long‐term unemployment during early working life among young adults	46,521 adolescents at the age of 25–28 years – Finland	Cohort	A very premature birth was associated with long‐term unemployment among men
Mikkonen et al. (2018)	The population impact of childhood health conditions on dropout from upper‐secondary education	How large a part of educational dropout is due to adverse childhood health conditions and to estimate the risk of dropout across various physical and mental health conditions	101,284 born in 1988–1995 followed for school dropout at ages 17 and 21 – Finland	Cohort	More than one fifth of educational dropout is attributable to childhood health conditions. Epilepsy, congenital heart disease, and severe infection showed strong associations to NEET. Cerebral palsy had the largest RR, but the estimation is uncertain due to small number of cases. Cancer and visual or hearing impairment exhibited large RRs at age 17 years, but at age 21 years their relationships with dropout status virtually vanished
Frobisher et al. (2017)	Employment status and occupational level of adult survivors of childhood cancer in Great Britain: The British Childhood Cancer Survivor Study	Detailed investigation of employment and occupation to be undertaken in a large population‐based cohort of childhood cancer survivors	10,257 5‐year survivors of childhood cancer – United Kingdom	Cohort	Obtaining employment and professional occupations is a problem for some groups of childhood cancer survivors, in particular for cranially irradiated CNS neoplasm and leukaemia survivors. Several factors were associated with employment status and occupational level such as childhood cancer type, radiotherapy, and medical conditions, for example, epilepsy
Glatz et al. (2017)	Association of anesthesia and surgery during childhood with long‐term academic performance	Association of anaesthesia and surgery before age 4 years with long‐term academic and cognitive performance indexed by school grades at age 16 years and IQ test scores at military conscription	33,514 children with 1 anaesthesia and surgery exposure before age 4 years and no subsequent hospitalization and 159,619 matched unexposed control children. Three thousand six hundred and forty children with multiple surgical procedures before age 4 years were also studied – Sweden	Cohort	Exposure to anaesthesia and surgery before age 4 years has a small association with later academic performance or cognitive performance in adolescence on a population level. – More vulnerable subgroups of children may exist, the low overall difference in academic performance after childhood exposure to surgery is reassuring
Díaz‐mendoza et al. (2015)	Analysis of employment rate and social status in young adults with childhood onset rheumatic disease in Catalonia	Determine the employment rate and social status of patients with childhood‐onset rheumatic disease attending a pediatric rheumatology transition unit	130 adolescents with childhood onset rheumatic disease mean age 22 years – Spain	Cross‐sectional	Some patients with childhood‐onset rheumatic disease encounter difficulties in their later social and working life. The time period needed to complete their studies tended to be longer, and incorporation into the workforce occurred at a later age
De Ridder et al. (2013)	Adolescent health and high school dropout: A prospective cohort study of 9000 Norwegian Adolescents (The Young‐HUNT)	School dropout in adolescents following chronic somatic disease, somatic symptoms, psychological distress, concentration difficulties, insomnia, or overweight	8950 school‐attending adolescents (13–21 years) rated their health in 1995–1997. High school dropout or completion, at 24 years – Norway	Cohort	All explored health dimensions were strongly associated with high school dropout. School dropout was strongly clustered within families
Homlong et al. (2013)	Can use of healthcare services among 15–16‐year‐olds predict an increased level of high school dropout? A longitudinal community study	Assess to which extent the family contributes to the association between health and school dropout	13,964 10th grade secondary school students – Norway	Cohort	Adolescents who seek help for conditions as serious illness, injury, headache, abdominal pain, neck pain, shoulder or back pain or pain in the extremities at certain healthcare services can be at risk of dropping out of high school later
Dommergues et al. (2010)	Current lifestyle of young adults after liver transplantation during childhood	Associations between healthcare seeking in 15–16‐year‐olds and high school dropout 5 years later	181 adolescents with a liver transplanted and mean age 21 years – France	Cross‐sectional	Delays in the academic level of the 75 still engaged in studies, according to the age group and gender.47% had not completed high schools at the time of the study. Women had reached higher levels of education than men. When only the 20‐ to 24‐year age group was considered, there were significant fewer women and men holders of the baccalaureate than in the reference population matched for age and gender
Fararouei et al. (2010)	Maternal Hb during pregnancy and offspring's educational achievement: A prospective cohort study over 30 years	Examine the association between maternal Hb levels during pregnancy and educational achievement of the offspring in later life	11,656 individuals born from singleton births (49% females) – Finland	Cohort	Low maternal Hb levels at the final stages of pregnancy are linked to the poorer educational achievement of the offspring
Bjerkedal et al. (2006)	Oppfølging av personer som fikk grunnstønad og/eller hjelpestønad som barn	Examine the health and social consequences of chronic illness in childhood	14,364 born 1967–76. Followed till the age of 27 years – Norway	Cohort	The following groups had a greater risk of low education and unemployment: Diabetes, neurological diseases, and disabilities
Calsbeek et al. (2006)	Disease characteristics as determinants of the labor market position of adolescents and young adults with chronic digestive disorders	Compare the employment status and disease burden in young adult patients with several chronic digestive disorders with healthy controls, and to determine whether labor participation depends on disease characteristics, such as type of diagnosis and burden of disease	622 patients (inflammatory bowel disease (IBD) *n* = 274, chronic liver diseases *n* = 78, congenital digestive disorders *n* = 104, food allergy *n* = 77, celiac disease *n* = 89), and a population‐based control group (*n* = 248), all age 15–24 – Netherland	Cohort	Patients with IBD or chronic liver diseases were found to have limited job prospects. In addition, gender and medication intake were found to be most determinative for a full‐time position
Kristensen et al. (2005)	Impact of life course determinants on work participation among young Norwegian men	To survey the health and social consequences of chronic illness in childhood	158,026 male singletons born in 1967–1971 data collected at age 29 – Norway	Cohort	Birthweight, childhood disease, and seven parental factors relating to income, disability, and family pattern, were independently associated with subsequent unemployment, each with population attributable risks ranging from 2% to 12%. Intellectual performance in young adult age, educational attainment, and marital status contributed substantially to the unemployment risk
Cooke (2004)	Health, lifestyle, and quality of life for young adults born very preterm	Association between birthweight and subsequent unemployment was mediated by intellectual performance at conscript	79 preterm and 71 term born children were assessed at age 19–22 years – United Kingdom	Cross sectional	Fewer preterm born were or had been in higher education, and some remained unemployed
Kristensen (2004)	Birthweight and work participation in adulthood	Association assessing to which extent it was influenced by circumstances concerning family background or disease in early life	308,829 singletons born in 1967–1971 who were national residents at age 29 – Norway	Cohort	Birthweight below the standardized mean was independently associated with unemployment at age 29, also in the normal birthweight range
Haberal et al. (2000)	Pediatric renal transplantation in Turkey: A review of 56 cases from a single center	Assess social and educational aspects of pediatric kidney transplantation (Tx) in Turkey, we retrospectively analyzed the results of 56 of these pediatric procedures performed at our center	56 adolescents with renal transplanted (48.2% female) – Turkey	Cohort	56% of the patients ended their education before high school

The majority of publications originate from Scandinavian countries, with a significant proportion of studies conducted within the last decade, reflecting an increasing emphasis on the topic during this period.

Most of the studies were cohort studies, with five designed as cross‐sectional (Cooke, 2004; Díaz‐mendoza et al., 2015; Dionisi‐Vici et al., 2022; Dommergues et al., 2010; Hövels‐Gürich et al., 2023).

The review identified multiple physical risk factors associated with NEET status. We used an inductive approach and categorized the identified risk factors into two primary domains: congenital conditions and birth‐related factors and health conditions during childhood and adolescence. The rationale for categorizing the identified risk factors into two primary domains stems from the distinct developmental stages and etiological pathways associated with each. Congenital conditions and birth‐related factors encompass health issues present at birth or arising during the perinatal period, which can significantly impact early development and long‐term health outcomes. On the other hand, health conditions during childhood/adolescence pertain to afflictions or disorders emerging during these developmental stages, influenced by various environmental, social, and genetic factors. By delineating risk factors into these domains, we aim to capture the unique characteristics and temporal dynamics of health determinants influencing the NEET status across different life stages, facilitating targeted interventions and policy initiatives.

Some studies investigated early life factors, examining life in utero, and how birth timing and birthweight influenced the risk of NEET status. Genetic influences like EA3 gene variants were linked to elevated unemployment rates (Ayorech et al., 2019) and congenital heart disease showed strong association with school dropout rates (Mikkonen et al., 2018).

Premature birth were consistently associated with unemployment (Cooke, 2004; Lallukka et al., 2019). However, one study found no associations of unemployment and being born preterm among adolescents born preterm, but those born at 39 weeks exhibited an increased risk compared to those born earlier (Murray et al., 2020). Low birthweight compared to the week of birth were also associated with unemployment (Kristensen, 2004, 2009). Rare conditions such as neonatal artery switch operation (Hövels‐Gürich et al., 2023) and cerebral palsy (Mikkonen et al., 2018; Pedersen et al., 2023) showed strong associations with school dropout rates and unemployment.

Moreover, chronic somatic diseases, including digestive disorders and type 1 diabetes, heightened the risk of NEET status during adolescents (Bjerkedal et al., 2006; Calsbeek et al., 2006; De Ridder et al., 2013; Kristensen et al., 2005; Liu et al., 2023). Adolescents with disabilities faced challenges in education attainment and employment (Abebe et al., 2019; Bjerkedal et al., 2006).

Cancer survivorship, particularly after cranially irradiated central nervous system (CNS) neoplasms and leukaemia, posed obstacles to employment later in life (Dionisi‐Vici et al., 2022; Frobisher et al., 2017); however, one study could not find an association between previous cancer diagnosis and unemployment (Baughan et al., 2023).

Epilepsy (Fleming et al., 2019b; Frobisher et al., 2017), liver and renal transplantation (Dommergues et al., 2010; Haberal et al., 2000), and exposure to anaesthesia early in life (Glatz et al., 2017) also showed a negative influence on educational outcomes.

Multimorbidity, particular among refugees, correlated with labor market marginalization, with varying risks associated with different diagnosis (Chen et al., 2021). Additionally, individual studies highlighted the elevated NEET risk associated with various conditions such as noncardiac comorbidity (Hövels‐Gürich et al., 2023), juvenile dermatomyositis (Boros et al., 2022), inherited retinal dystrophies (Kessel et al., 2022), musculoskeletal problems (Bania et al., 2019), severe infection in childhood (Mikkonen et al., 2018), rheumatic disease (Díaz‐mendoza et al., 2015), and neurological diseases (Bjerkedal et al., 2006). Common conditions like overweight (De Ridder et al., 2013) and musculoskeletal complaints were also linked to NEET status (Homlong et al., 2013).

However, some conditions, including asthma (Fleming, Fitton, et al., 2020), traumatic brain injury (Visnick et al., 2023), skin disorders (Fleming, McLay, et al., 2020), and celiac disease (Johansson et al., 2022), did not show significant associations with educational or employment status among young individuals.

## 4 DISCUSSION

The present review uncovers quantitative evidence regarding the physical risk factors associated with NEET among young Europeans aged 15–29 across Europe, thereby contributing to a comprehensive understanding of how specific illnesses impact children and young people in education and transitioning into the workforce. We will compare our results with other studies that have examined each of the identified risk factors. Additionally, we will incorporate insights from the latest scoping review on the topic (Rahmani et al., 2024). This scoping review differs from our study as it includes all risk factors and interventions to prevent NEET, and it included a global study population with both qualitative, quantitative, and mixed method designs. The scoping review critiques the varying classifications for the phenomenon of NEET. In our review, we utilized the OECD's criteria, focusing on youth aged of 15–29, which strengthens our research through rigorous selection. However, by excluding studies that included populations up to 30 or 35 years old, we may have missed important perspectives.

The absence of studies identified before the year 2000, despite the comprehensive search of data extracted after 1980, indicates a relatively emergence of focus on physical risk factors for NEET status within the field. Most of the study were cohort studies (84%) which typically provide stronger evidence of causal relationships due to their longitudinal nature. Five studies (16%) were cross‐sectional studies offering only a snapshot in time, making it difficult to infer causality. The mix of designs can complicate the synthesis of results and affect the overall conclusions.

The risk factors can be delineated into two primary domains: congenital conditions and birth‐related factors, and health conditions during childhood and adolescence.

### 4.1 Congenital conditions and birth‐related factors

The literature suggests that individuals born preterm, defined as birth before gestation week 37, may face increased challenges in achieving higher education and employment success, although the evidence supporting this assertion is not robust (Cooke, 2004). However, specific findings indicate that very preterm birth, occurring before gestation week 28, may significantly diminish the likelihood of employment for young men (Lallukka et al., 2019). These findings are supported by another study, which revealed that induction of labor was associated with lower academic performance at age 12 during lower secondary education, in contrast to cases where labor proceeded naturally (Burger et al., 2023). These findings underscore the nuanced relationship between gestational age and educational and vocational outcomes, emphasizing the need for further research to elucidate underlying mechanisms and guide targeted interventions for at‐risk populations.

Moreover, evidence suggests a potential influence of maternal factors on fetal development in utero. For instance, a study involving 11,656 participants found that low maternal haemoglobin levels during late pregnancy were associated with lower educational attainment in offspring (Fararouei et al., 2010). Additionally, infants with birth weights below the mean for gestational age appear to experience adverse effects on intellectual performance and employment prospects (Kristensen & Bjerkedal, 2004; Kristensen et al., 2005). Congenital heart disease have also shown a strong association to NEET status (Mikkonen et al., 2018). Another study have found that exposure to antiepileptic drugs in utero negatively affect the children's learning ability in primary school (Bech et al., 2018). A large study of 1,098,742 men found that perinatal diagnoses and congenital deformities was associated with lower IQ and that lower IQ was linked with unsuccessful educational and occupational achievement (Hegelund et al., 2018). These findings highlight the intricate interplay between maternal health, fetal development, and long‐term educational and vocational outcomes, warranting further investigation and targeted intervention strategies.

An intriguing new focus in research revolves around utilizing genetic factors as predictive indicators for identifying young individuals at risk of transitioning into the NEET category (not in employment, education, or training). One of the identified studies revealed that a one standard deviation increase in a genome‐wide polygenic score (EA3) was linked to a halving in the odds of having a full‐time job (Ayorech et al., 2019). There is a growing recognition that the prenatal environment significantly influences fetal development through epigenic modifications, which involve the activation or deactivation of genes in response to varying environmental influences. It is also understood that epigenic modifications may increase the risk of physical diseases in offspring (Neri & Edlow, 2016). This emerging approach represents a shift toward exploring the genetic predispositions and hereditary influences that may contribute to educational and vocational outcomes among young people. By examining genetic markers and variations, researchers aim to elucidate potential biological mechanisms underlying the NEET status and to develop predictive models that can identify at‐risk individuals early in their developmental trajectories. Furthermore, early life operations can significantly impact a child's development, as evidenced by studies showing that young individuals who underwent an artery switch operation in neonatal life were found to have double the unemployment rate compared to the average population (Hövels‐Gürich et al., 2023). Additionally, adolescents who were exposed to surgery or anaesthesia before the age of four also demonstrated significant effects on their development (Glatz et al., 2017).

### 4.2 Health conditions during childhood and adolescence

Transitioning to work‐life for childhood cancer survivors is a topic of interest; however, the evidence regarding this subject is inconclusive. A large cohort study comparing 1313 young individuals with a previous cancer diagnosis with 766,217 peers found no significant association (Baughan et al., 2023). Conversely, three other studies reported a negative impact on occupation 6 month after leaving school and a strong association with NEET status (Dionisi‐Vici et al., 2022; Frobisher et al., 2017; Mikkonen et al., 2018). Research has demonstrated that severe illnesses experienced during childhood significantly reduce the likelihood of young individuals pursuing further education after completing compulsory schooling or obtaining employment. This phenomenon has been observed in various conditions, including type 1 diabetes combined with psychiatric disorders, cerebral palsy, traumatic brain injury, autoimmune diseases, celiac disease, inherited retinal dystrophies, multimorbidity, skin disorders, musculoskeletal problems, asthma, disabilities, use of antiepileptic medication, rheumatic diseases, chronic somatic diseases, chronic liver diseases, and neurological disorders. The detrimental effects of maternal obesity on fetal programming in utero have been documented, with significant negative consequences for offspring neurodevelopmental outcomes. Research by Neri and Edlow indicates that maternal obesity or excessive weight gain during is associated with cerebral palsy, diabetes, and lower IQ in childhood with maternal obesity or excessive weight gain during pregnancy mediated inflammatory environment in utero (Neri & Edlow, 2016). These discoveries emphasize the significant influence of childhood illness on educational and vocational achievements, underscoring the necessity for tailored interventions and assistance for those affected. Nonetheless, absenteeism from school itself is linked to the failure to complete high school, particularly if it occurs at the start or end of the secondary school period (Keppens, 2023). Distinguishing whether the increased risk of NEET stems from the physical condition itself, as seen in severe brain injury cases, or from absenteeism, as may be more prevalent in skin disorders, poses a challenge. A study from Canada found no association between health‐related disabilities, defined as conditions preventing or limiting participation in school, play, sports, or any other activity typical for someone of the same age. The researchers suggest that the lack of association may be due to the primarily physical nature of these disabilities rather than cognitive impairments (Flynn & Tessier, 2012). Hence, it's imperative to acknowledge the impact of physical health on a child's cognitive development from prenatal stages through childhood and youth. Initiatives should be undertaken to support children who miss significant school due to treatment and recovery from illnesses.

A study from Peru found that demographic and background characteristics, such as academic performance and parental qualifications, are strong predictors of successful labor market transitions. In contrast, life‐limiting health conditions or disabilities demonstrated only weak predictive power regarding labor market outcomes (Dorsett & Lucchino, 2014). This suggests that, although our identified studies reported a risk associated with various physical conditions, other factors might have a stronger impact on the young individuals NEET status. The scoping review revealed that various factors, including socioeconomic, environmental, individual, school, and work characteristics, all influence young individuals' trajectories into further education or work (Rahmani et al., 2024). These findings underscores the importance of considering these factors in future studies on physical risk factors for NEET status. Moreover, the OECD (Eurofound et al., 2012) demonstrated that individuals facing disabilities or health challenges are 40% more likely to be NEET than their peers in good health. This highlights the critical need for understanding specific conditions that increase the risk of NEET, enabling early interventions to prevent NEET.

### 4.3 Limitations

Despite the thoroughness of this review, there are limitations worth noting. First, while extensive efforts were made to locate and classify studies systematically, there remains a risk of overlooking relevant research due to the selection of search terms and databases. Furthermore, excluding certain languages may limit the diversity and comprehensiveness of the literature review, potentially overlooking valuable insights and perspectives present in the studies written in the nonincluded languages. Second, the diversity of outcomes examined—ranging from unemployment to disability pensioning, NEET status, and education dropout—introduces complexity in determining the specific impact of physical variables on each outcome. Furthermore, our study's design prevents the calculation of effect size estimates yet provides a thorough understanding of the existing literature and identifies areas where knowledge gaps exist.

## 5 CONCLUSION

In conclusion, it is evident that varying health conditions during childhood and adulthood present obstacles to completing secondary school and entering the job market. Therefore, there should be a particular focus on children facing emerging health challenges from the fetal life and during childhood and adolescence, assisting them in staying abreast of their studies, providing specialized attention to their schooling needs postrecovery, and supporting them in maintaining academic attainment. However, this review highlights a scarcity of evidence regarding the impact of various health conditions on the NEET status of young individuals, indicating the need for further research into the effects of short‐ and long‐term illnesses.

## CONFLICT OF INTEREST STATEMENT

The authors declare no conflict of interest.

## ETHICS STATEMENT

The present study (review) did not fulfill the criteria and thus did not require formal approval from the ethical board.

## Data Availability

The search strategies from all databases are shown in the Supporting Information.

## APPENDIX 1

**Table:**

Database	Interface	Number of references	Date
Medline	Ovid	16,146	February 21, 2023
Embase	Ovid	16,934	February 21, 2023
PsycInfo	Ovid	5971	February 21, 2023
Scopus	Elsevier	12,075	February 21, 2023
All		51,126	
After duplicate removal in Covidence		33,323	

Embase

Database: Embase <1974 to 2023 January 30> Search Strategy:

‐‐‐‐‐‐‐‐‐‐‐‐‐‐‐‐‐‐‐‐‐‐‐‐‐‐‐‐‐‐‐‐‐‐‐‐‐‐‐‐‐‐‐‐‐‐‐‐‐‐‐‐‐‐‐‐‐‐‐‐‐‐‐‐‐‐‐‐‐‐‐‐‐‐‐‐‐‐‐‐

1 NEET.ti,ab,kw,kf. (234)
2 (NEETS or Hikikomori*).ti,ab,kw,kf. (270)
3 (Noneducat* or non educat*).ti,ab,kw,kf. or exp disadvantaged population/(1049)
4 unemploy*.ti,ab,kw,kf. or Unemployment/(37420)
5 (Socially excluded or truant* or economic* inactiv*).ti,ab,kw,kf. (757)
6 ((No or out or "not" or dropout or disconnect* or attach* or detach*) adj2 (job* or work* or school* or educat* or employ* or training*)).ti,ab,kw,kf. (103035)
7 (youth* or adolescen* or juvenile* or early adulthood).ti,ab,kw,kf. (630714)
8 (young adj2 (adult* or person* or individual* or people* or population* or man or men or wom#n)).ti,ab,kw,kf. (306640)
9 Adolescent/(1718167)
10 Young Adult/(485970)
11 7 or 8 or 9 or 10 (2423334)
12 1 or 2 or 3 or 4 or 5 or 6 (140206)
13 11 and 12 (22130)
14 review.pt. (3006963)
15 exp "review"/(3059107)
16 exp controlled clinical trial/(952153)
17 14 or 15 or 16 (4237273)
18 ((opportunit* or disconnect*) adj1 (youth or adolescent* or juvenile* or early adulthood or (young adj2 (adult* or person* or individual* or people* or population* or man or men or wom#n)))).ti,ab,kw,kf. (139)
19 13 or 18 (22249)
20 19 not 17 (20148)
21 limit 20 to (yr="2000 ‐Current" and (danish or english or norwegian or swedish)) (16934)

PsycInfo

Database: APA PsycInfo <1806 to January Week 4 2023> Search Strategy:

‐‐‐‐‐‐‐‐‐‐‐‐‐‐‐‐‐‐‐‐‐‐‐‐‐‐‐‐‐‐‐‐‐‐‐‐‐‐‐‐‐‐‐‐‐‐‐‐‐‐‐‐‐‐‐‐‐‐‐‐‐‐‐‐‐‐‐‐‐‐‐‐‐‐‐‐‐‐‐‐

1 NEET.ti,ab. (159)
2 (NEETS or Hikikomori*).ti,ab. (212)
3 (Noneducat* or non educat*).ti,ab. (326)
4 unemploy*.ti,ab. (16738)
5 exp Unemployment/or exp Disadvantaged/(13439)
6 (Socially excluded or truant* or economic* inactiv*).ti,ab. (957)
7 ((No or out or "not" or dropout or disconnect* or attach* or detach*) adj2 (job* or work* or school* or educat* or employ* or training*)).ti,ab. (52093)
8 (youth* or adolescen* or juvenile* or early adulthood).ti,ab. (352575)
9 (young adj2 (adult* or person* or individual* or people* or population* or man or men or wom#n)).ti,ab. (112803)
10 exp Adolescent Development/(64631)
11 1 or 2 or 3 or 4 or 5 or 6 or 7 (77784)
12 exp Disadvantaged/(8566)
13 11 or 12 (77784)
14 8 or 9 or 10 (428618)
15 13 and 14 (11137)
16 exp "Systematic Review"/or exp "Literature Review"/(23718)
17 exp clinical trials/(13446)
18 ((opportunit* or disconnect*) adj1 (youth or adolescent* or juvenile* or early adulthood or (young adj2 (adult* or person* or individual* or people* or population* or man or men or wom#n)))).ti,ab. (187)
19 15 or 18 (11286)
20 16 or 17 (37087)
21 19 not 20 (11246)
22 limit 21 to ((danish or english or norwegian or swedish) and yr="2000 ‐Current") (8381)
23 limit 22 to ("0100 journal" or "0110 peer‐reviewed journal" or "0120 non‐peer‐reviewed journal" or "0130 peer‐reviewed status unknown" or "0500 electronic collection") (5971)

Medline

Database: Ovid MEDLINE(R) and Epub Ahead of Print, In‐Process, In‐Data‐Review & Other Non‐Indexed Citations and Daily

<1946 to January 30, 2023>

Search Strategy:

‐‐‐‐‐‐‐‐‐‐‐‐‐‐‐‐‐‐‐‐‐‐‐‐‐‐‐‐‐‐‐‐‐‐‐‐‐‐‐‐‐‐‐‐‐‐‐‐‐‐‐‐‐‐‐‐‐‐‐‐‐‐‐‐‐‐‐‐‐‐‐‐‐‐‐‐‐‐‐‐

1 NEET.ti,ab,kw,kf. (206)
2 (NEETS or Hikikomori*).ti,ab,kw,kf. (200)
3 (Noneducat* or non educat*).ti,ab,kw,kf. (344)
4 unemploy*.ti,ab,kw,kf. or Unemployment/(25554)
5 (Socially excluded or truant* or economic* inactiv*).ti,ab,kw,kf. (643)
6 ((No or out or "not" or dropout or disconnect* or attach* or detach*) adj2 (job* or work* or school* or educat* or employ* or training*)).ti,ab,kw,kf. (78529)
7 (youth* or adolescen* or juvenile* or early adulthood).ti,ab,kw,kf. (508075)
8 (young adj2 (adult* or person* or individual* or people* or population* or man or men or wom#n)).ti,ab,kw,kf. (231432)
9 Adolescent/(2200530)
10 Young Adult/(1003568)
11 7 or 8 or 9 or 10 (2943862)
12 1 or 2 or 3 or 4 or 5 or 6 (103646)
13 11 and 12 (22968)
14 review.pt. or exp "review"/(3121840)
15 systematic review.pt. or exp "systematic review"/(218609)
16 controlled clinical trial.pt. or exp controlled clinical trial/(677259)
17 14 or 15 or 16 (3896228)
18 ((opportunit* or disconnect*) adj1 (youth or adolescent* or juvenile* or early adulthood or (young adj2 (adult* or person* or individual* or people* or population* or man or men or wom#n)))).ti,ab,kw,kf. (113)
19 13 or 18 (23060)
20 19 not 17 (20904)
21 limit 20 to (yr="2000 ‐Current" and (danish or english or norwegian or swedish)) (16146)

Scopus

PUBYEAR > 1999 AND ((((TITLE‐ABS (neet OR neets OR Hikikomori* OR noneducat* OR "non educat*" OR unemploy* OR "Socially excluded" OR truant* OR "economic* inactiv*")) OR (TITLE‐ABS (((no OR out OR "not" OR dropout OR disconnect* OR attach* OR detach*) PRE/2 (job* OR work* OR school* OR educat* OR employ* OR training*))))) AND ((TITLE‐ABS ((young W/2 (adult* OR person* OR individual* OR people* OR population* OR man OR men OR wom#n)))) OR (TITLE‐ABS (youth* OR adolescen* OR juvenile* OR "early adulthood")))) OR (TITLE‐ABS ((opportunit* OR disconnect*) W/1 (youth OR adolescent* OR juvenile* OR "early adulthood" OR (young W/2 (adult* OR person* OR individual* OR people* OR population* OR man OR men OR wom#n)))))) AND NOT (TITLE (review* OR trial*)) AND (EXCLUDE (DOCTYPE, "re") OR EXCLUDE (DOCTYPE, "ch") OR EXCLUDE (DOCTYPE, "bk") OR EXCLUDE (DOCTYPE, "cr")) AND (LIMIT‐TO (LANGUAGE, "English") OR LIMIT‐TO (LANGUAGE, "Danish") OR LIMIT‐TO (LANGUAGE, "Norwegian") OR LIMIT‐TO (LANGUAGE, "Swedish"))

Updated search: January 15, 2024.

**Table:**

Database	Interface	Number of references	Date
Medline	Ovid	779	January 15, 2024
Embase	Ovid	1443	January 15, 2024
Psycinfo	Ovid	200	January 15, 2024
Scopus	Elsevier	1243	January 15, 2024
All		3665	
After duplicate removal in Covidence		2535	
